# Sexual Victimization Disclosure: A Cluster Analysis Approach to Understanding Victimization Experiences in Disclosers and Non-Disclosers

**DOI:** 10.3390/ijerph182211919

**Published:** 2021-11-13

**Authors:** Kaitlin Walsh Carson, Sara Babad, Mahathi Kosuri, Mikell Bursky, Victoria Fairchild, Usha Barahmand, Elissa J. Brown, Valentina Nikulina

**Affiliations:** 1 Psychology Department, The Graduate Center, City University of New York, New York, NY 10016, USA; sara.babad@qc.cuny.edu (S.B.); mahathi.kosuri@qc.cuny.edu (M.K.); mikell.bursky@qc.cuny.edu (M.B.); victoria.fairchild@qc.cuny.edu (V.F.); valentina.nikulina@qc.cuny.edu (V.N.); 2 Psychology Department, Queens College, City University of New York, New York, NY 11367, USA; usha.barahmand@qc.cuny.edu; 3 Child HELP Partnership, St. John’s University, Queens, New York, NY 11439, USA; browne@stjohns.edu

**Keywords:** sexual victimization, PTSD, disclosure status, cluster analysis

## Abstract

Research has used cluster analysis to identify clusters, or groups, of sexual victimization survivors who share similar assault experiences. However, researchers have not investigated whether disclosure status is a key component of the survivors’ experience. The current study identified two clusters among 174 disclosing and non-disclosing sexual victimization survivors. Cluster One (*n* = 74) included an incapacitated assault by a lesser-known perpetrator and disclosure of the event. Cluster Two (*n* = 100) included a verbally instigated assault by a well-known perpetrator and nondisclosure of the event. Follow up independent *t*-tests revealed that women in Cluster One had significantly higher depression and posttraumatic stress disorder (PTSD) symptoms than women in Cluster Two. Results support prior research identifying clusters of victimization based on assault characteristics and suggest that disclosure status is a key variable in the recovery process. Specific implications for clinicians, policy makers, and the community are discussed.

Sexual victimization includes attempted or completed rape, forced penetration, sexual coercion, and/or unwanted sexual contact and is a major public health concern in the United States, particularly among emerging adult women (i.e., late teens through 29-years-old) [1,2]. At least 43% of women report experiencing some form of sexual violence in their lifetime, with 81% of completed or attempted rape survivors reporting that this victimization occurred prior to or during emerging adulthood [2]. Sexual victimization survivors are at an increased risk for the development of depression and posttraumatic stress disorder (PTSD) [3,4,5]. Specifically, among college-attending female rape survivors, depression prevalence rates range from 9.1–13.1% [6]. Similarly, research has consistently reported elevated PTSD rates in survivors of interpersonal violence, including rape and other forms of sexual assault compared with other types of trauma exposure (e.g., natural disaster, car accident) [7,8]. Specifically, 17.4% of rape survivors and 11.0% of sexual assault survivors met criteria for PTSD, compared with lifetime prevalence rates ranging from 1.3% to 8.8% across all types of trauma [7].

Despite extensive research into sexual assault survivors, an overwhelming majority of the studies is focused on “disclosers” or women who come forward to disclose sexual victimization and identify as survivors [9,10,11,12]. Other studies do not attend to disclosure and nondisclosure, leaving out this key part of the survivor’s experience related to symptomology, help-seeking behaviors, and recovery [9]. The *non-disclosers* make-up a significant minority of survivors, 25%, whose experiences have been understudied and underrepresented in the sexual assault literature. Therefore, the current study builds on previous work [11] aiming to replicate previous findings on clusters of groups of survivors and to also investigate how disclosure status clusters with assault characteristics to result in differences in PTSD and depression symptomology.

## 1. Emerging Adulthood

The developmental time period spanning from age 18 years old through 29 years old is dubbed emerging adulthood [13]. It is marked by transitions, exploration, and instability, which contribute to vulnerability to sexual victimization [1]. In fact, 81% of female survivors of completed or attempted rape and 71% of female survivors of sexual violence, physical violence, or stalking by an intimate partner report that the incident occurred prior to or during emerging adulthood [2]. Given the elevated rates of unwanted sexual experiences reported by female emerging adults, it is imperative to study this developmental period. Moreover, the United States Census Bureau estimates that currently 50% of all emerging adults are enrolled in college, a rate that is higher than at any other time in history [14,15]. These estimates are even higher among females, compared with males [14,16], highlighting the importance of examining sexual victimization experiences in emerging adult, college-attending females. Research has demonstrated that among emerging adults, sexual victimization experiences and rates vary by sexual orientation and gender identity [17], and therefore, studies that do not take these factors into consideration present a partial view of sexual victimization.

### 1.1. Patterns of Assault Characteristics

Prior research [18] has identified specific assault characteristics of the victimization experience that help explain the disparity in the rates of development of psychopathology across sexual victimization survivors: assault tactic (e.g., verbal pressure, authority, incapacitation via drugs/alcohol, physical force), assault event (e.g., sex play, sex acts, attempted intercourse, intercourse), and perpetrator identity (e.g., stranger, acquaintance, romantic acquaintance, spouse, relative). Additionally, research has linked assault characteristics with different levels of PTSD symptoms, conceptualizing assault tactics on a continuum ranging from lowest severity (i.e., verbal coercion) to highest severity (i.e., physical force), corresponding with lowest PTSD symptoms to highest PTSD symptoms, respectively [19]. Similarly, the type of event that occurs is also viewed on a continuum ranging from lowest severity (i.e., sex play) to highest severity (i.e., completed intercourse) corresponding, respectively, with lowest PTSD symptoms to highest PTSD symptoms [19]. Some studies have identified common associations between pairs of individual assault characteristics. Specifically, survivor alcohol consumption during the assault is reported to be associated with a less violent assault tactic compared with assaults where the survivor does not consume alcohol [11,20,21,22]. This is potentially due to the perpetrator’s ability to use less force against the survivor when she is incapacitated [11]. Additionally, survivors of alcohol-related assaults are less likely to know the perpetrator than survivors of alcohol-unrelated assaults [22].

Notably, many of these assault characteristics do not occur in isolation, but rather, they tend to cluster together. Therefore, examining assault characteristics individually may fail to adequately conceptualize the survivor’s experience. For example, attempted intercourse via verbal pressure from a significant other is a different experience than attempted intercourse via physical force from a relative and may have a differential impact on the survivor’s overall recovery process.

Only one study known to the authors used cluster analysis to integrate assault characteristics and create a cluster variable to describe the experiences of sexual victimization survivors [11]. This sample consisted of 877 community women, ages 18–69 (*M* = 34.5, *SD* = 12.0), who had previously disclosed their unwanted sexual experience. The analysis yielded three clusters, which the researchers described as: (1) High-Violence Assaults, (2) Alcohol-Related Assaults, and (3) Moderate Sexual-Severity Assaults. The High-Violence cluster emerged as the group with the most violent assault tactics and most severe assault events (i.e., completed rape). Survivors in the Alcohol-Related assault encompassed alcohol-related assaults (i.e., 94% of perpetrators were drinking and 85% of survivors were drinking prior to the assault) and had comparable assault event severity (i.e., completed rape) with the High-Violence assaults, but with only moderately severe assault tactics. The Moderate Sexual-Severity assault cluster contained the lowest levels of assault event severity and tactic violence. At Time 1, Alcohol-Related assaults had lower PTSD symptoms than High-Violence assaults, and both clusters had higher PTSD symptoms than Moderate Sexual-Severity assaults. However, when assessed one year later, Alcohol-Related assaults did not differ in PTSD symptoms from High-Violence assaults, but both clusters continued to have more severe symptoms than Moderate Sexual-Severity assaults. These results suggest that composite variables can provide a better understanding of mental health outcomes for sexual victimization survivors.

Although this study has provided substantial evidence for the existence of distinct clusters of assault characteristics that distinguish *disclosers* of sexual victimization, there remains a gap in the literature regarding understanding the experiences of *non-disclosers* and discerning whether they can be differentiated from *disclosers* [9,11]. Survivors’ disclosure status (i.e., whether or not the survivor discussed their experience with another individual) is a key factor that has the potential to impact post-assault coping and recovery [9]. The current study aims to fill these gaps by investigating whether disclosure status also clusters with the specific aforementioned assault characteristics (e.g., assault tactic, assault event type, perpetrator identity) to further explain the experiences of disclosing and non-disclosing survivors and their mental health sequelae.

### 1.2. Disclosure Status

Disclosure of a traumatic event involves discussing the experience either verbally or in a written form [12,23]. Common treatment approaches with trauma survivors involve encouraging the survivor to disclose their experience through the development of a trauma narrative [24,25]. This narrative is expressed repeatedly by the survivor to another individual through verbal, written, or artistic means with the goal of gradually reducing the survivor’s distress surrounding the experience [23,24,25,26]. Survivors typically experience negative mood and physical symptoms (e.g., elevated heart rate) while disclosing and soon after the disclosure [23,26]. In the long-term, however, disclosure is associated with positive physical and psychological consequences [23,26]. Nonetheless, multiple factors can complicate the disclosure experience for sexual assault survivors. A range of variables are known to influence the outcome of sexual assault disclosure, including: (1) contextual factors (e.g., private or social setting for disclosure), (2) discloser characteristics (e.g., gender, age, culture, personality, attachment style, coping), (3) disclosure characteristics (e.g., voluntariness, modality, level of detail, timeliness), (4) perceived reaction to disclosure (e.g., positive or negative), and (5) any history of childhood sexual abuse [12]. The disclosure process for sexual victimization survivors is complex and likely influences the recovery of emerging adult survivors.

As discussed above, most research on survivors of sexual assault has focused exclusively on the experiences of disclosers. Very few studies [9,27] have examined the experiences of non-disclosers or compared the experiences of both groups (disclosers and non-disclosers) together. These studies have reported that nearly 25% of female sexual victimization survivors keep this experience to themselves. Through understanding similarities and differences in the experiences of both disclosers and non-disclosers, insightful information can be gained that can aid in implementing improved preventive and therapeutic interventions for this population.

### 1.3. Associations between Disclosure Status, Assault Characteristics, and Symptomology

Recently, some researchers have investigated patterns of associations between disclosure status (i.e., whether or not the survivor has previously disclosed their experience to another individual), assault characteristics, and psychopathology. Women who disclosed their experience were more likely to report drug and alcohol use during the assault than those who did not disclose [9]. Women who were in a more distant relationship with the perpetrator (e.g., acquaintance) were more likely to disclose their experience than those who reported a more intimate relationship with the perpetrator (i.e., romantic acquaintance) [9,28].

There are discrepancies in the literature when examining differences in symptomology across disclosers and non-disclosers. Ahrens et al. (2010) found that nondisclosure is associated with increased depression and PSTD symptoms, whereas Carson et al. (2019) found no overall differences in symptomology between disclosers and non-disclosers. One possible explanation for this contradiction is that disclosure status in these studies was examined as an isolated variable. Considering a range of sexual victimization characteristics in combinations can highlight the nuances of the survivors’ experiences and help explain symptomology [11]. Additionally, the differences in methodology utilized by these two studies may also explain this discrepancy. Ahrens and colleagues (2007) invited sexual victimization survivors to participate in a confidential face-to-face interview, whereas Carson and colleagues (2019) conducted an online study in which participants disclosed their experiences to a computer, and researchers did not advertise the study as specifically targeting sexual assault survivors.

#### Current Study

This study was designed to investigate whether disclosure status is a key component in the mental health outcomes of sexual assault survivors. In the current study, data were collected online, which allowed for increased anonymity compared with face-to-face interviews [29,30]. Online methodology allowed us to reach an important and understudied group of survivors who had not previously disclosed information about their victimization experience. The aims of the current study were to: (1) replicate the findings of Peter-Hagene and Ullman (2015) among 174 disclosing and non-disclosing emerging adult female survivors, and (2) examine how disclosure status clustered with assault characteristics to better understand differences in PTSD and depression symptomology.

## 2. Method

### 2.1. Participants and Procedures

Six hundred and four undergraduate women from two urban universities, one in New York City, NY and one in Miami, FL, participated in this online study in 2008 and 2009. Participants selected this study from a range of options available to Psychology 101 students. Participants were informed that the data were being collected as part of the principal investigator’s (last author’s) dissertation research. The study goal was described as understanding the experiences of women who have been through “tough times” and the association between disclosure of negative sexual experiences and psychological functioning. Participants were told that the online study takes approximately 40 min. The risks and benefits were described, and participants provided informed consent by reading the consent document and indicating whether they are willing to continue with the survey. The researchers used the latest versions of the instruments available to them at the time of data collection in 2008 and 2009. Standardized instructions that were provided by the developers of the measures were given to the participants when they completed each measure, except when responding to the Social Reaction Questionnaire, which was adapted to assess the first disclosure experience (see the methods section below). Of the 604 students, 37% (*n* = 221) disclosed a history of sexual victimization (i.e., kissing, fondling, petting, attempted intercourse, completed intercourse). A total of 174 of women, ranging in age from 18 to 29 years (*M* = 20.5; *SD* = 2.4), were included in the current study. Across all 174 participants, 75.3% (*n* = 131) previously disclosed their experience and 24.7% (*n* = 43) had not disclosed their experience to anyone prior to participating in the current study. The majority of the participants, 81.6% (*n* = 142), were from Miami. Participants in this study were a diverse group of undergraduate women; 57.5% (*n* = 100) identified as Caucasian, 9.8% (*n* = 17) as African American, 7.5% (*n* = 13) as multiracial, 2.9% (*n* = 5) as Asian, and 22.4% (*n* = 39) as “Other.” Of the 39 participants who identified as “Other,” 94.7% (*n* = 36) identified their race as Hispanic. Ethnicity was also examined, with 57.5% of all participants identifying as Hispanic and 29.3% identifying as Non-Hispanic. Participants in the current study self-identified as women; data were not collected on sexual orientation or gender identity.

Participants selected the current study from other available studies offered by the universities and were compensated with course credit. Informed consent was obtained from all participants involved in the study. The study was conducted according to the guidelines of the Declaration of Helsinki and approved by the Institutional Review Board of St. John’s University (protocol codes #0108-061 and #2015-0304). The institutional review boards approved all study procedures. Women who chose to participate in the survey first completed a demographics questionnaire, followed by the *Sexual Experiences Survey* (SES) [18]. Women who disclosed an experience of sexual victimization completed additional measures. Upon completion of the survey, participants were debriefed, thanked, and provided with mental health resources.

### 2.2. Measures

#### 2.2.1. The Demographics Questionnaire

Questions about participants’ age, race, and ethnicity were included. The race and ethnicity categories included in the current study were derived from the United States 2010 Census categories (Race and Ethnicity, n.d.). Participants identified their racial background from a list of available categories and were able to select more than one category (i.e., African American or Black, American Indian or Alaskan Native, Asian or Asian American, Caucasian or White, Native Hawaiian or other Pacific Islander, and Other). Participants identified their ethnic background by selecting Hispanic or Non-Hispanic.

#### 2.2.2. The Sexual Experiences Survey (SES)

The Sexual Experiences Survey (SES) [18] is a self-report measure of unwanted sexual experiences. Participants reported whether they experienced a variety of unwanted sexual experiences ranging in increasing severity from “sex play (fondling, kissing, or petting)” to “sex (vaginal, anal, or oral intercourse).” Participants also reported on the assault tactics that led to their experience ranging in increasing severity from “because you were overwhelmed by a man’s continual arguments and pressure” to “because a man threatened or used some degree of physical force.” Data were also collected on the survivor−perpetrator relationship (stranger, acquaintance, casual date, romantic acquaintance, spouse, relative, or other) and disclosure status (i.e., whether participant discussed this experience prior to the online study). This survey has been used with a large number of college students [18,31] and sexual victimization survivors [32]. This measure also has strong validity with college-attending women. The Pearson correlation between a woman’s level of victimization as reported on the SES and the level of victimization identified during an interview with a psychologist was 0.73 (*p* < 0.001) [33].

#### 2.2.3. Beck Depression Inventory, Second Edition (BDI-II)

The Beck Depression inventory, Second Edition (BDI-II) [34] is a 21-item self-report assessment that measures a range of depressive symptoms, including, sadness, anhedonia, hopelessness, guilt, and changes in sleep and appetite. Standard administration and scoring guidelines were followed. Participants choose a response for each item that best reflects how they have been feeling in the past two weeks from four possible options representing levels of severity of a symptom. Each item is scored on a 4-point scale (0 to 3) with possible total scores ranging from 0 to 63. Higher scores indicate higher levels of depressive symptomology. The BDI-II is a widely used measure with strong reliability and validity [35,36], including with survivors of sexual victimization [37,38]. In the current study, Cronbach’s alpha was 0.93, which is similar to figures reported by studies with similar populations [37]. The presence of a depression diagnosis was also assessed. Survivors reported an average of 10.41 (*SD* = 10.0) symptoms, which indicates minimal depression [34]. Based on the standard cut-off score guidelines, 64% of participants did not meet criteria for depression and 36% met criteria for depression, with 16% of the total sample endorsing mild depression, 14% endorsing moderate depression, and 6% endorsing severe depression.

#### 2.2.4. The PTSD Symptom Scale (PSS)

The PTSD Symptom Scale (PSS) [39] is a 17-item self-report measure assessing participants’ symptoms of PTSD over the past two weeks. This measure is based on DSM-IV-TR criteria [40]. Symptom items are rated on a Likert scale of frequency and severity ranging from 0 (Not at all) to 3 (5 or more times per week/very much/almost always) with possible total scores ranging from 0 to 51. For the purposes of this study, a diagnostic determination of PTSD (yes/no) was made, and the total symptomology severity was calculated based on standard scoring guidelines [39]. Higher scores indicate higher levels of PTSD symptomology. Participants who reported experiencing more than one sexual victimization experience completed this assessment in reference to the most severe sexual victimization experience reported on the SES. The PSS has been reported to possess adequate validity and reliability with survivors of sexual victimization [27,41]. In the current study, PSS had a Cronbach’s alpha of 0.91, which is consistent with prior research with a similar population [27,41], and survivors reported on average 8.43 (*SD* = 8.8) symptoms, which is slightly lower than figures reported by studies with similar populations [27]. Additionally, 46% of the current sample met DSM-IV-TR criteria for PTSD, which is consistent with prior research [11,40].

## 3. Data Analysis

In pursuance of Aim 1 and to identify clusters of assault characteristics and disclosure status, a two-step cluster analysis was conducted. Prior to including variables as clustering criterion, chi-square analyses were used to confirm that the various assault characteristics were not independent of each other and to limit Type I error [42]; see Table 1. As demonstrated in prior research, the preliminary bivariate analyses indicated that assault tactic (pressure, authority, drugs/alcohol, physical force) varied by perpetrator identity and disclosure status. Additionally, assault event type (e.g., sex play, sex acts, attempted intercourse, completed intercourse) varied by assault tactic. After confirming that the assault characteristics are associated with each other, a two-step cluster analysis was performed in SPSS v. 25 with perpetrator identity (stranger, acquaintance, casual date, romantic acquaintance, spouse, relative, other), assault tactic (pressure, authority, drugs/alcohol, physical force), assault event (sex play, sex acts, attempted intercourse, completed intercourse), and disclosure status (disclosers, non-disclosers) entered as cluster criterion variables. Schwarz’s Bayesian Clustering Criterion (BIC) method with a log-likelihood distance measure was used. To increase the representativeness of the identified clusters, criterion variables were limited to variables previously established as having a significant impact on survivor recovery [11]. The analysis yielded two clusters. Follow-up chi-square analyses were conducted to determine differences in criterion variables across clusters.

In accordance with Aim 2 and to identify PTSD and depression symptom differences across clusters, two independent *t*-tests were run with cluster membership entered as the independent variable and total PTSD symptoms and total depression symptoms entered as the dependent variables. Additionally, chi-square analyses were used to identify the association between cluster membership and PTSD and depression diagnoses.

## 4. Results

### 4.1. Aim One: Identify Sexual Victimization Clusters

Results of the two-step cluster analysis yielded two clusters (see Table 2). Cluster One (“distant relationship, incapacitation/force, predominantly disclosers”) consisted of 74 women and Cluster Two (“intimate relationship, verbal coercion, predominantly non-disclosers”) consisted of 100 women. Results of the chi-square analyses revealed that women who were categorized in Cluster One were more likely to disclose the experience, more likely to have a distal relationship with the perpetrator (i.e., stranger, acquaintance, other), and the sexual victimization experience was more likely to be instigated by drugs/alcohol or physical force and consisted of more sex acts and attempted intercourse. Women who were categorized into Cluster Two were less likely to disclose their experience, more likely to have been in an intimate relationship with the perpetrator, and the victimization experience was more likely to be instigated by verbal pressure or authority and consisted of more sex play and completed intercourse. See Table 2.

### 4.2. Aim Two: Investigating Symptom Differences across Clusters

Significant differences in total PTSD and depression symptomology were identified between Cluster One and Cluster Two (see Table 3). Specifically, women in Cluster One had higher depression (*M* = 12.46) and PTSD (*M* = 10.91) symptoms than the women in Cluster Two (*M* = 6.60; *M* = 8.91, respectively). Additionally, chi-square analyses revealed that 56.8% and 43.2% of women in Cluster One met criteria for PTSD and depression compared with 38% and 30% of women in Cluster Two, respectively. While there were significant differences in regard to PTSD diagnoses, no significant differences emerged regarding depression diagnosis across the two clusters.

## 5. Discussion

The goal of the current study was to identify groups of women with similar sexual victimization experiences by creating cluster variables that integrate assault characteristics and disclosure status to further understand the sexual victimization experiences of disclosing and non-disclosing emerging adult survivors. Overall, the current findings highlight the importance of including disclosure status to help clinicians and researchers better understand the experiences of emerging adult sexual victimization survivors. Furthermore, the two clusters identified in the current study can help to further explain disparities in PTSD and depression symptoms among disclosing and non-disclosing sexual victimization survivors.

Women in Cluster One, survivors who experienced an assault involving physical force as well as assaults involving alcohol, were more likely to be victimized by an acquaintance or stranger [11,43]. These assaults involving physical force, alcohol, and a less-known perpetrator were associated with higher rates and symptoms of PTSD, which is consistent with prior research [11,22]. Additionally, our findings suggest that women categorized in Cluster One were more likely to disclose their experience. These women’s experiences can be perceived to represent more “stereotypical” assaults and replicate prior literature that women with stereotypical assault experiences are more likely to come forward [27,28,44].

Cluster Two reflects a unique group of non-disclosing survivors whose experiences have not been characterized thoroughly in the literature. Because there is limited research on the experiences of non-disclosers, there is less of a precedent to compare the patterns of Cluster Two with. Cluster Two consisted of women who did not previously disclose their experience and who reported a more intimate relationship with the perpetrator, which is consistent with previously identified patterns for non-disclosers [9,28]. Given many of the women in Cluster Two reported an intimate relationship with the perpetrator, it is possible that this cluster of women represents a group of intimate partner violence (IPV) survivors. Thirty-eight percent of the women in Cluster Two met criteria for PTSD, and 30% met criteria for depression. These rates are lower than prevalence rates found in prior research for both PTSD and depression among IPV survivors [45]. This may reflect the fact that the women in the current study have not previously disclosed their experience and are, therefore, underrepresented in current research. Further examination of mental health outcomes and assault characteristics of non-disclosers of IPV will be important in order to better understand this particular group of sexual victimization survivors. Additionally, research has estimated that 18.3% of women in the United States experience sexual violence by an intimate partner in their lifetime [2]. Our findings suggest that current prevalence rates may underestimate the extent of this public health concern given the number of survivors not disclosing these experiences. Furthermore, it is possible that these women are experiencing additional forms of maltreatment from their partners (e.g., physical abuse, psychological abuse) and that the sexual violence is only one part of their experience [2]. In the future, it will be important to screen these women for various forms of abuse in order to provide appropriate services.

Our findings differed from prior research in several ways. Extant literature indicates that increased assault event severity is associated with increased PTSD symptoms [19]. In the current study, assault event type did not group by severity. Specifically, women in Cluster One were more likely to experience more moderate levels of assault event severity, including sex acts and attempted intercourse, and reported higher symptoms of PTSD and depression. Conversely, women in Cluster Two were more likely to experience sex play (least severe event) or completed intercourse (most severe event) and reported fewer symptoms of PTSD and depression. This difference in findings may be a function of examining the combined effects of disclosure status, perpetrator identity, assault tactic, and assault type together. Whereas prior research has examined assault event severity in isolation, the current study, in addition to examining the severity of the assault event, accounted for how well the survivor knew the perpetrator, whether or not the survivor disclosed the experience, and how severe the assault tactic was. Examining this combination of factors can help to better explain the survivors’ negative mental health sequelae.

Furthermore, many of the women in Cluster Two who experienced completed intercourse did not disclose their experience prior to the research study. Because the majority of prior research has focused exclusively on the experiences of disclosers of sexual victimization (finding assault event severity associated with increased PTSD symptoms), this subgroup of non-disclosing survivors is underrepresented in prior research. These results highlight the importance of increasing our efforts to include these survivors in research. Understandably, this group of survivors can be difficult to locate and difficult to engage in sexual victimization research as they are not discussing their experience. However, the current findings suggest that previously established conclusions regarding sexual victimization experiences and the subsequent recovery process (e.g., completed rape is associated with increased PTSD), may not be relevant for all sexual victimization survivors. Specifically, it is important to recognize that extant sexual victimization literature may not be generalizable to the 25% of survivors who do not share their experiences with anyone [9,27,46]. Including more non-disclosing women in sexual victimization research can help us to generalize findings to more survivors and develop more appropriate interventions.

## 6. Clinical Implications

The results of the current study highlight the importance of taking multiple assault and disclosure characteristics into consideration when attempting to understand a survivor’s experience. It is also important to note that the severity of the sexual event type did not directly relate to PTSD or depression symptomology.

Common approaches to treatment and interventions for trauma survivors often involve discussing details about the traumatic event [24,25]. Although this approach may be helpful in some instances, it is important to recognize that therapeutic approaches involving exposure to the traumatic experience through the development of a trauma narrative may not be the most relevant intervention for all survivors. The use of a trauma narrative in therapy is intended to help the survivor make sense of their experience and to serve as a type of exposure therapy. The trauma narrative is expressed repeatedly by the survivor through verbal, written, or artistic means with the goal of gradually reducing the survivor’s distress surrounding the experience [24,25]. However, this may not be appropriate for non-disclosing survivors who are experiencing symptoms of PTSD but are reluctant to share their victimization experience. Furthermore, when survivors report an intimate relationship with the perpetrator, it may be necessary and beneficial to also target known risk factors for intimate partner violence, such as couple conflict and poor interpersonal communication skills [47].

Over half of the women in the current study reported that they had a romantic relationship with the perpetrator, providing further support for research that has identified college-aged women as the age group at highest risk for experiencing intimate partner violence (IPV) [48]. Moreover, the majority of the women who engaged in an intimate relationship with the perpetrator had not disclosed the event. Campbell and colleagues (2009) argue that broad cultural attitudes regarding rape and societal rape myths are often internalized by survivors, impacting their response to and recovery from the trauma. One such rape myth that may be internalized by the current sample is that having unwanted or unconsented sex with a romantic partner (e.g., boyfriend or husband) is not considered sexual assault [49]. This may be one possible explanation as to their reason for not disclosing. Furthermore, studies have documented that men who internalize rape myths have a lower ability to correctly interpret consent during a sexual encounter [50]. These findings highlight the need to provide appropriate IPV screenings and universal preventions with emerging adult females. Findings also underscore the importance of working with college campuses to provide preventative psychoeducation regarding consent, sexual assault, and intimate partner violence for both emerging adult males and females. Individual universities throughout the United States that have begun to adopt such preventive measures have seen a subsequent decrease in sexual assault rates [51], highlighting the importance of integrating preventative educational measures at universities across the country.

Another common rape myth is that if completed intercourse did not occur, then the event cannot be considered sexual assault [49]. In the current study, a substantial proportion of the survivors of sexual victimization reported sex play as the sexual assault and also reported elevated PTSD and depression symptomatology (see Table 2 and Table 3). Educating survivors and the public about the different types of sexual assault events along with the subsequent negative mental health outcomes may encourage survivors to seek the support they need as well as help teach communities how to respond appropriately to disclosures of sexual victimization involving sex play, sex acts, or intercourse.

## 7. Strengths and Limitations

It is important to note that there are several limitations in the interpretation of these novel data. The sample recruited was comprised of undergraduates and is not necessarily representative of survivors of sexual victimization at a regional or national level. A multi-center study with individuals from the general population would improve the external validity of the study. A large percentage of the sample identified as Hispanic, disproportionate to the proportion of Hispanics in the general United States population. Although this may limit the generalizability of our results to the general population, these findings contribute to the limited research on sexual assault and disclosure within the Hispanic population [52]. Additionally, data on participants’ sexual orientation and gender identity were not collected. As such, the findings of the current study may not generalize to all survivors’ experiences of disclosure and non-disclosure. Of note, while research has shown that women who identify as lesbian, gay, bisexual, queer, or transgender (LGBTQ) are at an increased risk for sexual victimization, studies have also suggested that LGBTQ and heterosexual emerging adult sexual victimization survivors are equally likely to disclose their experiences to formal sources [17].

This study was conducted online and relied solely on retrospective self-report data, limiting findings to the perceptions of each individual participant. However, this is also a strength of the current study as online data collection allows for increased anonymity, which allowed us to capture experiences of non-disclosers [29,30]. No data were excluded following a data quality assurance check to screen for implausible demographic combinations (i.e., race, ethnicity, place of birth) [53]. Additionally, this online survey was restricted to undergraduate students who received course credit, which reduced the likelihood of women participating multiple times and eliminated participants who search the internet for paid studies [53]. As these data were collected in 2008 and 2009, an older version of the *Sexual Experiences Survey* was used; however, this measure still has demonstrated strong validity in college age women [18,31].

It is important to note that the obtained data are cross-sectional, and causality cannot be inferred. Furthermore, the cross-sectional design implies an evolution of the clusters over time, and whether clusters are stable could not be determined. In addition, the prevalence and nature of sexual victimization is changing, and it is unclear how stable the obtained clustering is over time. Additionally, although these data provide a new perspective and understanding on clustering among women survivors of sexual victimization whose disclosures were collected in 2008 and 2009, the data may not be easily generalizable to all present-day survivors, particularly when considering the recent “#MeToo” social movement, which encourages survivors of sexual victimization to disclose their experience. However, these results are still likely to be representative of the experiences of survivors, as many of the women who disclosed during the #MeToo movement reported sexual victimization experiences that occurred in the past. The obtained findings are still generalizable to many of the survivors who continue to keep this experience to themselves. Furthermore, apart from disclosure status, severity of the assault event, and degree of familiarity with the perpetrator, all of which contributed to the clustering, other variables such as length of acquaintance and personality traits such as intelligence, resilience, and extroversion, which may impact the clustering, were not assessed. Independent replication of the clusters was not performed. Finally, it must be noted that a cluster analytical approach does not allow us to understand the etiology of the clusters.

## 8. Future Directions

Despite these limitations, this is the only study known to the research team that integrates disclosure status with assault characteristics to further understand sexual victimization experiences. With the increase in the societal focus on sexual victimization, it is important to draw upon research to educate the public about these experiences in order for all survivors to receive the support and help they may need. Future research will benefit from the continued inclusion of non-disclosers of sexual victimization to further understand the experiences of this understudied group. Future studies should continue to examine nondisclosure among different populations, including IPV survivors, individuals who identify as LGBTQ, males, and different cultural and ethnic groups. Finally, the field would benefit from additional longitudinal studies to elucidate the direction of the relationship between victimization experiences, disclosure status, and survivor well-being.

## 9. Conclusions

The current study identified two clusters of female sexual victimization survivors. The first cluster is defined by more stereotypical sexual assault experiences, a higher likelihood of disclosure, and is marked by higher levels of mental health difficulties. The second cluster represents victimization perpetrated within more intimate relationships and is associated with reduced likelihood of disclosure. These results suggest that disclosure is a key variable to examine when investigating sexual victimization experiences. Overall, the current findings highlight the importance of considering a range of variables including the type of sexual assault instigator (e.g., verbal, force, drugs/alcohol) and disclosure status to help clinicians and researchers better understand the experiences of emerging adult sexual victimization survivors as well as survivors of intimate partner violence.

## Figures and Tables

**Table 1:** Preliminary chi-square analyses among criterion variables.

Variables	Assault Tactic	Assault Event	Perpetrator Identity	Disclosure Status
Assault Tactic	---	χ^2^ (9) = 120.51 ***	χ^2^ (18) = 75.13 ***	χ^2^ (3) = 10.07 *
Assault Event	---	---	χ^2^ (18) = 25.62	χ^2^ (3) = 1.26
Perpetrator Identity	---	---	---	χ^2^ (6) = 21.25 **
Disclosure Status	---	---	---	---

Note: *** *p* < 0.001; ** *p* < 0.01; * *p* < 0.05.

**Table 2:** Criterion Variables by Cluster Membership.

	Cluster One (*n* = 74)	Cluster Two (*n* = 100)	Chi-Square
Perpetrator			χ^2^ (6, 174) = 19.31 **
Stranger	**2.7%**	0%	
Acquaintance	**35.1%**	19%	
Casual Date	10.8%	**14%**	
Romantic Acquaintance	33.8%	**52%**	
Spouse	0%	**5%**	
Relative	4.1%	**6%**	
Other	**13.5%**	4%	
Assault Tactic			χ^2^ (3, 174) = 174.00 ***
Pressure	0%	**93%**	
Authority	0%	**7%**	
Drugs/Alcohol	**40.5%**	0%	
Physical Force	**59.5%**	0%	
Assault Event			χ^2^ (3, 174) = 93.75 ***
Sex Play	6.8%	**60%**	
Sex Acts	**8.1%**	0%	
Attempted Intercourse	**52.7%**	0%	
Completed Intercourse	32.4%	**40%**	
Disclosure Status			χ^2^ (1, 174) = 5.00 *
Disclosers	**83.8%**	69%	
Non-Disclosers	16.2%	**31%**	

Note: Criterion variables by cluster membership; *** *p*< 0.001; ** *p* < 0.01; * *p* < 0.05. % refers to the percentage of participants in each cluster that reported the characteristic (e.g., 93% of cluster one participants experienced pressure as the assault tactic); bolded percentages are greater than expected.

**Table 3:** PTSD and depression differences across clusters.

Outcome	Cluster One	Cluster Two	*t* (df)	Cohen’s *d*
PSS Total, M (SD)	10.91 (9.67)	6.60 (7.69)	3.16 (135.46) **^	0.49
BDI-II Total, M (SD)	12.46 (10.74)	8.91 (9.08)	2.35 (173) *	0.36
			Chi-Square (df)	Phi Coefficient
PTSD Diagnosis (% meet criteria)	56.8%	38%	6.02 (1) *	0.19
Depression Diagnosis (% meet criteria)	43.2%	30%	3.25 (1)	0.14

Note: ** *p* < 0.01; * *p* < 0.05. ^ adjusted *t* and *p*-values as Levene’s test for equal variances was significant.

## Data Availability

Data for this study are not available for sharing for ethical and confidentiality reasons. At the time of data collection, participants were not made aware, or consented to, the possibility of data sharing. For any questions, please contact the corresponding author.
